# Association Between Thyroid Function Parameters and Plasma Branched-Chain Amino and Keto Acids in Patients With Euthyroid Type 2 Diabetes Mellitus

**DOI:** 10.1155/jdr/2540444

**Published:** 2025-07-02

**Authors:** Xinghua Cai, Yuanying Xu, Jie Gao, Huihui Zhang, Jun Lu, Tao Lei

**Affiliations:** ^1^Shanghai Putuo Central School of Clinical Medicine, Anhui Medical University, Shanghai, China; ^2^The Fifth School of Clinical Medicine, Anhui Medical University, Hefei, China; ^3^Department of Endocrinology, Putuo Hospital, Shanghai University of Traditional Chinese Medicine, Shanghai, China; ^4^School of Medical and Life Sciences, Chengdu University of Traditional Chinese Medicine, Chengdu, China

**Keywords:** branched-chain amino acids, branched-chain keto acids, thyroid, Type 2 diabetes mellitus

## Abstract

**Objectives:** The objective of the study is to determine the association between thyroid function parameters and plasma levels of branched-chain amino acids (BCAAs) and their metabolites, branched-chain keto acids (BCKAs), in patients with euthyroid Type 2 diabetes mellitus (T2DM).

**Methods:** We conducted a cross-sectional study among 357 participants with euthyroid T2DM. Plasma BCAAs and BCKAs were measured using enzyme-linked immunosorbent assay (ELISA), while four thyroid function parameters, including triiodothyronine (T3), thyroxine (T4), free triiodothyronine (FT3), and free thyroxine (FT4), were determined by an automatic biochemical analyzer. The variance inflation factor (VIF) was used to investigate variable collinearity. Pearson correlation and multivariate linear regression analysis were used to establish the relationship between BCAAs or BCKAs and thyroid function parameters.

**Results:** The means of T3, T4, FT3, and FT4 were different among the three groups divided by BCAA tertiles (all *p* < 0.05), and the means of T3 and FT3 were different among the three groups divided by BCKA tertiles (all *p* < 0.01). Pearson correlation analysis indicated a positive relationship between all the thyroid function parameters and BCAA levels (*p* < 0.05), while a statistically significant inverse relationship was established between T3 and FT3 levels and BCKA levels (*p* < 0.01). Collinearity analysis demonstrated no collinearity among the variables (tolerance: 0.501~0.922; VIF: 1.084~1.995). Multivariate linear regression analysis showed that thyroid function parameters were significantly associated with BCAAs (coefficients: 0.042 to 4.614, *p* < 0.05) and BCKAs (coefficients: −0.200 to −0.156, *p* < 0.01) after adjusting for other potential confounding factors.

**Conclusions:** Thyroid function parameters positively correlated with plasma BCAAs but negatively correlated with plasma BCKAs in patients with euthyroid T2DM. However, further prospective studies are needed to explore potential causal relationships and verify these findings.

## 1. Introduction

Branched-chain amino acids (BCAAs: valine, leucine, and isoleucine) play important physiological roles in regulating food intake, protein synthesis, metabolism, and aging [1]. BCAAs, which are synthesized in plants and microorganisms but not in animals, must be obtained through diet [2]. Maintaining optimal plasma levels of BCAAs is essential for maintaining overall physiological equilibrium in the human body. Lower levels of BCAAs can impair normal growth processes and disrupt homeostasis [3]. Conversely, higher levels of BCAAs have been associated with a variety of metabolic disorders, including insulin resistance, Type 2 diabetes mellitus (T2DM), obesity, thyroid dysfunction, cardiometabolic disease, and cancer epidemics [4–6].

In recent years, the association between BCAAs and thyroid diseases has increasingly attracted attention. Thyroid dysfunction can manifest as either an overproduction (hyperthyroidism) or underproduction (hypothyroidism) of thyroid hormones. Recent research by Lijuan et al. has revealed a reduction in plasma BCAAs when transitioning from a hyperthyroid to a euthyroid state [7]. Additionally, other studies have found that plasma levels of BCAAs are relatively lower in individuals with hypothyroidism compared to those with normal thyroid function [8]. It follows that higher thyroid functional status is positively associated with elevated plasma BCAA levels. It is essential to note that earlier investigations have suggested a possible association between BCAAs and the onset of diabetes and cardiovascular disease [2, 9], and thyroid dysfunction is a common complication of T2DM [10, 11]. T2DM and thyroid diseases share the common characteristic of dysregulated metabolism.

BCAAs and thyroid dysfunction are both contributors to metabolic abnormalities in T2DM. However, studies on the relationship between plasma BCAAs and thyroid function in patients with T2DM, particularly those with normal thyroid function, remain limited and warrant further investigation. Therefore, in the present study, we aim to investigate the association between the levels of BCAAs and their metabolic byproducts, branched-chain keto acids (BCKAs), and thyroid hormone levels (including triiodothyronine (T3), thyroxine (T4), free triiodothyronine (FT3), and free thyroxine (FT4) [12]) in euthyroid T2DM patients, which may contribute to the development of novel diagnostic and therapeutic strategies for this population.

## 2. Materials and Methods

### 2.1. Study Population

From January 2022 to August 2023, we recruited 357 patients with confirmed T2DM from the electronic medical records of the endocrinology department in Shanghai Putuo District Central Hospital. The diagnostic criteria for T2DM were based on the American Diabetes Association in 2021 [13]. Patients were excluded if found to show any of the following conditions: (1) Type 1 diabetes mellitus, (2) severe hepatic or renal insufficiency, (3) medications affecting thyroid function (antithyroid drugs, thyroid hormone replacement, lithium, or iodine), (4) pregnancy or puerperium, (5) malignant tumors, and (6) patients with hyperthyroidism or hypothyroidism.

The protocol was approved by the Shanghai University of Traditional Chinese Medicine Ethics Committee Affiliated Putuo Hospital (PTEC-R-2022-82(Y)-1). Signed informed consent was acquired from each participant.

### 2.2. Blood Sampling

After at least 8 h of fasting, each participant had 5 mL of blood extracted from their median cubital veins in the morning, which was then stored in 10-mL glass tubes at 4°C within 12 h. Following 15 min of centrifugation to separate plasma from whole blood, the samples were stored at −80°C for further analysis.

### 2.3. Anthropometric Measurements

During the hospitalization period, we conducted extensive anthropometric measurements on all patients. Firstly, we employed standard methods to measure height and weight and calculated the body mass index (BMI, kilogram/square meter) by dividing the weight (in kilograms) by the square of the height (in meters). Additionally, we measured their blood pressure using a mercury sphygmomanometer while the patients were supine, with two readings taken for each individual and the average calculated. Hypertension (HTN) was defined as systolic blood pressure (SBP) ≥ 140 mmHg and/or diastolic blood pressure (DBP) ≥ 90 mmHg [14], current use of antihypertensive drugs, or self-reported history of HTN.

### 2.4. Clinical Laboratory Measurements

The disease history and drug treatment of all patients were recorded on admission. Biochemical indexes, including thyroid-stimulating hormone (TSH), T3, T4, FT3, FT4, fasting plasma glucose (FPG), fasting insulin (F-INS), and lipid profiles, were measured by the Beckman Coulter AU5800 automatic biochemical analyzer in Brea, California, United States. A high-performance liquid chromatography analyzer, HLC-723G1, was used to detect glycosylated hemoglobin type A1C (HbA1c). We evaluated the homeostasis model assessment of insulin resistance (HOMA-IR), an index of insulin resistance, using the formula: HOMA‐IR = FPG (millimoles/liter) × F‐INS (micro‐international units/milliliter)/22.5 [15]. Enzyme-linked immunosorbent assay (ELISA) kits were utilized to detect BCAAs (supplier: Abcam, catalog number: ab83374, sensitivity: 0.2 nmol/well) and BCKAs (supplier: Fu Sheng Biologicals, China, catalog number: A137748, sensitivity: 0.25 *μ*g/L). Based on BCAA levels (Table 1), the participants were divided into three groups: Tertile 1 (BCAA levels ≤ 2.022 mmol/L, *n* = 119), Tertile 2 (2.022 mmol/L < BCAA levels ≤ 2.610 mmol/L, *n* = 119), and Tertile 3 (BCAA levels > 2.610 mmol/L, *n* = 119). Similarly, based on BCKA levels (Table 2), the participants were also divided into three groups: Tertile 1 (BCKA levels ≤ 108.77* μ*mol/L, *n* = 119), Tertile 2 (108.77 *μ*mol/L < BCKA levels ≤ 138.40 *μ*mol/L, *n* = 119), and Tertile 3 (BCKA levels > 138.40* μ*mol/L, *n* = 119).

### 2.5. Statistical Analysis

All statistical analyses were carried out using SPSS Version 22.0 and GraphPad Prism Version 9.0.0. Continuous variables were presented as median (interquartile range) and categorical variables as proportions. The Kruskal–Wallis test was used to test the differences in medians, and the *χ*^2^ test was used to examine differences for categorical data. The general linear model was employed to evaluate the associations between thyroid function parameters (TSH, T3, T4, FT3, and FT4) and tertiles of plasma BCAAs and BCKAs, with the least significant difference (LSD) method used for post hoc between-group comparisons. The relationship between BCAAs or BCKAs and thyroid function parameters was analyzed using Pearson correlation analysis. Multiple linear regression analysis was utilized to examine the association of BCAAs and BCKAs with thyroid function parameters considering other parameters (Tables 3 and 4). All anthropometric parameters in Table 4 were converted to *Z*-scores. Before including independent variables in the study, a collinearity test was conducted. A variance inflation factor (VIF) greater than 1 and closer to 1 indicates less multicollinearity, whereas a VIF less than 0.1 or a VIF greater than 2 suggests the presence of multicollinearity. Two-sided *p* values less than 0.05 were considered statistically significant.

## 3. Results

### 3.1. Description of Participant Baseline Characteristics

This study included a total of 357 adult individuals with T2DM. Based on their levels of BCAAs or BCKAs, participants were divided into three groups (tertiles, T1–T3), with 119 individuals in each group. Table 1 summarizes the clinical characteristics of T2DM patients with different BCAA levels. The thyroid function parameters (T3 (*p* < 0.001), T4 (*p* < 0.01), FT3 (*p* < 0.001), and FT4 (*p* < 0.05)), triglyceride (TG) levels (*p* < 0.001), and the prevalence of current statin use (*p* < 0.05) were lower in the first tertile of BCAA levels compared to the second and third tertiles. In addition, the prevalence of current insulin use was higher in the first tertile compared to the second and third tertiles of BCAA levels (*p* < 0.01).

Table 2 summarizes the clinical data of T2DM patients with different BCKA levels (tertiles, T1–T3). The thyroid function parameters (T3 (*p* < 0.001) and FT3 (*p* < 0.01)), lipid profile levels (total cholesterol (TC) (*p* < 0.01), high-density lipoprotein cholesterol (HDL-C) (*p* < 0.001), low-density lipoprotein cholesterol (LDL-C) (*p* < 0.01)), and HOMA-IR (*p* < 0.01) were higher in the first and second tertiles of BCKA levels, compared to the third tertile, while F-INS levels (*p* < 0.01) and the prevalence of current insulin use (*p* < 0.05) were lower.

### 3.2. Thyroid Function Parameters Among Different BCAA or BCKA Groups

We utilized bar graphs to demonstrate the relationship between different levels of BCAAs and BCKAs with thyroid function. As shown in Figure S1, based on the three tertiles categorization of BCAAs, we observed significant differences in the mean T3 levels among the three groups of samples (*p* < 0.001), with corresponding mean values (nanomoles/liter) of 1.40 (first tertile), 1.61 (second tertile), and 1.58 (third tertile). Among these three groups, the mean values of T4 (nanomoles/liter) were 100.2, 109.7, and 110.2, respectively (*p* < 0.01). Similarly, significant differences were found in the mean values of FT3 (picomoles/liter) and FT4 (picomoles/liter) (*p* < 0.001).

We observed significant differences in mean T3 levels among the three groups of samples categorized based on three quartiles of BCKAs (*p* < 0.001). The corresponding mean values (nanomoles/liter) were 1.60 (first tertile), 1.56 (second tertile), and 1.43 (third tertile). Similarly, the mean values of FT3 (picomoles/liter) differed significantly among the three groups, with values of 4.69, 4.60, and 4.33 (*p* < 0.05). Nevertheless, there were no significant differences in T4 and FT4 among groups categorized by BCKA tertiles.

### 3.3. Relationship Between Thyroid Function Parameters and BCAAs or BCKAs

Figure S2 shows the correlation between BCAAs and T3 (*r* = 0.131, *p* < 0.05), T4 (*r* = 0.132, *p* < 0.05), FT3 (*r* = 0.171, *p* < 0.01), and FT4 (*r* = 0.182, *p* < 0.001) by Pearson's correlation analysis. However, BCKAs showed a significant inverse correlation with two thyroid function parameters: T3 (*r* = −0.190, *p* < 0.001) and FT3 (*r* = −0.164, *p* < 0.01).

Considering the sample size, collinearity diagnostic results, and clinical significance of this study, variables with VIF greater than 2 were eliminated. The multivariate regression analysis included BCAAs, BCKAs, age, gender, diabetes mellitus (DM) duration, HTN, SBP, DBP, smoking status, drinking status, FPG, HbA1c, HDL-C, BMI, and current medication use (including insulin, metformin, alpha-glucosidase inhibitor (AGI), dipeptidyl peptidase 4 inhibitor (DPP4i), sodium–glucose cotransporter 2 inhibitor (SGLT2i), statin, and angiotensin-converting enzyme inhibitor/angiotensin II receptor blocker (ACER/ARB)) as independent variables (tolerance: 0.501~0.922; VIF: 1.084~1.995). We examined the association between BCAAs or BCKAs and thyroid function parameters using multivariate regression analysis in Table 3. A covariate adjustment was not made in Model 1, which showed a positive association between four thyroid function parameters (T3, T4, FT3, and FT4) and BCAAs (the coefficients ranged from 0.050 to 3.427, all *p* < 0.05). After controlling for age and gender (Model 2) and further adjusting for duration of diabetes, HTN, SBP, DBP, smoking status, drinking status, FPG, HbA1c, HDL-C, BMI, and current medication use (including insulin, metformin, AGI, DPP4i, SGLT2i, statin, and ACER/ARB) (Model 3), BCAA levels remained significantly and positively associated with T3, T4, FT3, and FT4 (the coefficients ranged from 0.042 to 0.050 for T3, from 3.427 to 4.614 for T4, from 0.115 to 0.184 for FT3, and from 0.430 to 0.589 for FT4, all *p* < 0.05). However, two thyroid function parameters (T3 and FT3) were inversely associated with BCKAs (the coefficients ranging from −0.005 to −0.002, all *p* < 0.05).

### 3.4. Relationship Between T3 *Z*-Scores, FT3 *Z*-Scores, and BCKA *Z*-Scores

Considering that regression coefficients with absolute values less than 0.1 may be influenced by different units of measurement, we transformed anthropometric parameters into *Z*-scores. Through multivariate regression analysis (Table 4), we found that BCKA *Z*-scores showed consistent inverse associations with both T3 *Z*-scores (the coefficients ranging from −0.200 to −0.192, all *p* < 0.001) and FT3 *Z*-scores (the coefficients ranging from −0.186 to −0.156, all *p* < 0.01).

## 4. Discussion

BCAAs and their keto acid derivatives (BCKAs) have emerged as potential biomarkers and metabolic regulators in T2DM. T2DM is one of the most rapidly increasing metabolic disorders, characterized by chronic hyperglycemia, insulin resistance, and dyslipidemia [16]. Elevated plasma BCAA levels have been consistently observed in diabetic patients and animal models [17–19], with clinical studies linking them to insulin resistance (positive association with HOMA-IR) [20, 21] and impaired F-INS secretion [22]. In our study, although F-INS levels were higher and HOMA-IR lower in the first tertile of BCAA levels compared to the second and third tertiles, these differences did not reach statistical significance (*p* > 0.05), potentially due to limited sample size. Notably, BCKAs showed a paradoxical positive association with F-INS but an inverse correlation with HOMA-IR, suggesting distinct roles of BCAA catabolites in glucose metabolism.

Beyond glycemic control, BCAAs may interact with broader metabolic risk factors. Previous studies associate elevated BCAA levels with smoking, HTN, hyperlipidemia, metabolic syndrome, and high BMI [9, 17, 23]. Consistently, we observed significant differences in lipid profiles (TC, TG, HDL-C, and LDL-C) across BCAA or BCKA tertiles. Pharmacological interventions may also modulate BCAA metabolism: rosuvastatin and insulin therapy have been reported to alter BCAA levels [24, 25]. In our study, the Tertile 1 BCAA group had a higher proportion of insulin users but lower statin use compared to Tertiles 2–3, highlighting potential drug–metabolite interactions.

T2DM and thyroid disorders are common metabolic diseases that frequently coexist, with bidirectional pathophysiological links [26, 27]. Clinical evidence indicates that T2DM patients are more prone to thyroid disorders than the general population [11, 28, 29], and even within the euthyroid range, higher TSH correlates with metabolic dysregulation [30, 31] and worsened diabetic complications [32]. Given that both T2DM and thyroid hormones modulate mitochondrial function [33, 34], we hypothesized that BCAA metabolism—a mitochondrial-dependent process—could serve as a mechanistic bridge between these conditions.

To investigate the potential regulatory effects of BCAAs on thyroid hormones while eliminating confounding by overt thyroid dysfunction, we rigorously enrolled euthyroid T2DM patients (normal T3, T4, FT3, FT4). Our key findings include the following: (1) Plasma BCAAs positively correlated with T3, T4, FT3, and FT4 levels (all *p* < 0.05), independent of confounders in multivariate regression. This aligns with Krishnamurthy et al.'s report of leucine-associated T4 elevation [35]; (2) BCKAs exhibited an inverse association with T3 and FT3, suggesting catabolite-specific effects.

Mechanistically, BCAAs may regulate thyroid function via mTOR signaling. Leucine, a potent mTOR activator [36], could enhance hypothalamic–pituitary–thyroid (HPT) axis activity, increasing hormone synthesis [37]. Conversely, thyroid hormones modulate mitochondrial BCAA oxidation by regulating the branched-chain *α*-keto acid dehydrogenase (BCKDH) complex [33, 34], creating a potential feedback loop. Our observation of divergent BCAA/BCKA associations supports this duality: elevated BCAAs may reflect mTOR-driven thyroid stimulation, while accumulated BCKAs could indicate impaired BCKDH-mediated degradation.

This study demonstrates significant correlations of BCAAs and BCKAs with thyroid hormone levels, suggesting that BCAA metabolism may regulate thyroid function in T2DM patients. These findings provide novel insights into the mechanisms of metabolic disorders in T2DM and, more importantly, establish a translational foundation for developing metabolic intervention strategies targeting thyroid dysfunction by elucidating the interplay between BCAA metabolism and thyroid function. Specifically, clarification of this association mechanism may yield the following clinical benefits: (1) providing potential metabolic biomarkers for early risk assessment of thyroid dysfunction in T2DM patients and (2) enabling novel therapeutic approaches for improving thyroid function through dietary modulation or targeted pharmacological interventions on BCAA metabolic pathways.

However, it is important to note that our observed associations should be interpreted as preliminary evidence requiring verification in studies with more comprehensive covariate assessment, including dietary patterns, physical activity levels, and other metabolic parameters. Although the current cross-sectional design cannot establish causality, multicenter prospective cohort validation with rigorous control of potential confounders could render these findings valuable evidence for the precision diagnosis and treatment of T2DM complicated by thyroid dysfunction.

## 5. Conclusions

This study on euthyroid patients with T2DM demonstrates that thyroid function parameters are positively correlated with plasma BCAAs but negatively correlated with plasma BCKAs. Prospective studies are guaranteed to confirm whether increasing levels of BCAAs or reducing levels of BCKAs may help to relieve low thyroid hormone states in the context of T2DM management.

## Figures and Tables

**Table 1:** Characteristics of study participants with different plasma BCAA levels.

**Variables**	**BCAA tertiles**			**p** ** value**
	**Tertile 1 (** **n** = 119**)**	**Tertile 2 (** **n** = 119**)**	**Tertile 3 (** **n** = 119**)**	
Gender, male	68 (57.0)	71 (60.0)	77 (65.0)	0.700
Age, years	64.8 (61.0–70.0)	63.4 (60.0–71.0)	61.5 (57.0–69.0)	0.239
DM duration, months	141 (27–240)	125 (12–216)	137 (27–240)	0.426
Current smoker, %	18 (15.0)	35 (29.0)	30 (25.0)	0.145
Current drinkers, %	17 (14.0)	25 (21.0)	31 (26.0)	0.495
BMI, kg/m^2^	23.4 (20.5–25.5)	24.2 (21.3–26.1)	24.4 (21.6–27.1)	0.090
T3, nmol/L	1.4 (1.2–1.6)	1.6 (1.3–1.8)	1.6 (1.4–1.8)	< 0.001
T4, nmol/L	100 (81–117)	109 (96–123)	110 (97–123)	0.003
FT3, pmol/L	4.3 (3.8–4.7)	4.6 (4.2–5.0)	4.7 (4.3–5.1)	< 0.001
FT4, pmol/L	15.9 (14.5–17.8)	16.1 (14.4–17.8)	16.9 (15.2–18.2)	0.020
TSH, *μ*IU/mL	3.2 (0.8–1.8)	2.3 (1.1–2.3)	2.1 (1.1–2.6)	0.054
HTN, %	63 (53.0)	76 (64.0)	61 (51.0)	0.125
SBP, mmHg	135 (125–143)	132 (120–140)	135 (124–141)	0.167
DBP, mmHg	81.5 (76–90)	81.2 (76–88)	81.3 (77–90)	0.462
FPG, mmol/L	7.7 (5.7–9.5)	8.3 (5.8–9.9)	8.6 (6.8–10.1)	0.121
HbA1C, %	9.8 (7.8–10.9)	9.8 (7.9–11.1)	9.6 (7.9–11.0)	0.453
F-INS, pmol/L	69.7 (32.3–123.9)	53.0 (29.4–105.0)	49.1 (28.4–106.2)	0.161
HOMA-IR, mmol/L⁣^∗^mU/L	20.9 (8.7–35.9)	21.0 (10.0–39.9)	21.9 (10.9–39.5)	0.199
TC, mmol/L	4.66 (3.89–5.41)	4.97 (3.88–5.78)	4.86 (3.67–5.80)	0.454
TG, mmol/L	1.34 (0.93–1.60)	2.05 (1.12–1.93)	2.01 (1.09–2.25)	< 0.001
HDL-C, mmol/L	1.16 (0.95–1.35)	1.14 (0.92–1.29)	1.21 (0.88–1.25)	0.503
LDL-C, mmol/L	2.97 (2.24–3.57)	3.05 (2.39–3.56)	3.11 (2.38–3.82)	0.654
BCAA, mmol/L	1.5 (1.1–1.9)	2.3 (2.2–2.5)	3.3 (2.8–3.6)	< 0.001
BCKA, *μ*mol/L	127 (98–153)	125 (103–140)	120 (99–142)	0.039
Current medication use, %				
Insulin	79 (66.0)	65 (55.0)	62 (52.0)	0.003
Metformin	43 (36.0)	55 (46.0)	46 (39.0)	0.391
AGI	43 (36.0)	39 (33.0)	50 (42.0)	0.490
DPP4i	33 (28.0)	23 (19.0)	29 (24.0)	0.537
SGLT2i	29 (24.0)	46 (39.0)	51 (43.0)	0.075
Statin	46 (39.0)	62 (52.0)	62 (52.0)	0.011
ACEI/ARB	39 (33.0)	57 (48.0)	31 (26.0)	0.173

*Note:* Data are presented as median (IQR) for continuous variables and number (proportion) for category variables. Differences in medians were examined using the Kruskal–Wallis test among three groups. Differences in proportions were tested using the chi-square test.

Abbreviations: ACER/ARB, angiotensin-converting enzyme inhibitor/angiotensin II receptor blocker; AGI, alpha-glucosidase inhibitor; BCAAs, branched-chain amino acids; BCKAs, branched-chain keto acids; BMI, body mass index; DBP, diastolic blood pressure; DPP4i, dipeptidyl peptidase-4 inhibitor; F-INS, fasting insulin; FPG, fasting plasma glucose; FT3, free triiodothyronine; FT4, free thyroxine; HbA1C, glycosylated hemoglobin, type A1C; HDL-C, high-density lipoprotein cholesterol; HOMA-IR, homeostatic model assessment of insulin resistance; HTN, hypertension; LDL-C, low-density lipoprotein cholesterol; SBP, systolic blood pressure; SGLT2i, sodium–glucose cotransporter 2 inhibitor; T3, triiodothyronine; T4, thyroxine; TC, total cholesterol; TG, triglyceride; TSH, thyroid-stimulating hormone.

**Table 2:** Characteristics of study participants with different plasma BCKA levels.

**Variables**	**BCKA tertiles**			**p** ** value**
	**Tertile 1 (** **n** = 119**)**	**Tertile 2 (** **n** = 119**)**	**Tertile 3 (** **n** = 119**)**	
Gender, male	73 (61.0)	71 (60.0)	73 (61.0)	0.982
Age, years	63.7 (60.8–69.0)	63.7 (60.0–70.0)	62.3 (58.0–72.0)	0.403
DM duration, months	129 (5.3–198)	128 (24–240)	146 (33–240)	0.901
Current smoker, %	35 (29.0)	21 (18.0)	26 (22.0)	0.129
Current drinkers, %	27 (23.0)	17 (14.0)	15 (13.0)	0.203
BMI, kg/m^2^	23.5 (21.4–25.4)	23.9 (21.5–26.7)	25.0 (21.5–27.2)	0.095
T3, nmol/L	1.6 (1.4–1.8)	1.5 (1.3–1.8)	1.4 (1.2–1.6)	< 0.001
T4, nmol/L	106 (90–121)	108 (94–124)	104 (87–121)	0.360
FT3, pmol/L	4.7 (4.3–5.0)	4.5 (4.2–4.9)	4.3 (4.0–4.7)	0.003
FT4, pmol/L	16.6 (14.8–18.5)	16.1 (14.5–17.8)	16.0 (14.9–17.6)	0.592
TSH, *μ*IU/mL	1.8 (0.9–2.4)	3.0 (1.1–2.0)	2.7 (1.0–2.6)	0.689
HTN, %	61 (51.0)	74 (62.0)	66 (55.0)	0.686
SBP, mmHg	136 (125–141)	132 (120–140)	134 (120–145)	0.148
DBP, mmHg	83 (76–90)	81 (77–87)	82 (77–90)	0.453
FPG, mmol/L	8.2 (6.0–9.7)	8.2 (5.7–9.6)	8.1 (5.9–10.0)	0.410
HbA1C, %	9.6 (7.8–10.9)	9.7 (8.1–10.9)	9.9 (7.9–11.3)	0.462
F-INS, pmol/L	51.7 (29.0–81.3)	59.3 (32.8–120.1)	78.9 (40.8–140.3)	0.004
HOMA-IR, mmol/L⁣^∗^mU/L	24.6 (9.8–53.2)	22.9 (13.5–39.5)	16.7 (8.1–31.9)	0.007
TC, mmol/L	5.2 (4.2–6.3)	4.6 (3.9–5.4)	4.6 (3.7–5.2)	0.001
TG, mmol/L	1.4 (1.0–1.9)	1.8 (1.0–2.2)	2.2 (1.2–2.1)	0.114
HDL-C, mmol/L	1.2 (1.0–1.4)	1.1 (1.0–1.2)	1.2 (0.9–1.2)	< 0.001
LDL-C, mmol/L	3.3 (2.6–4.0)	3.0 (2.3–3.6)	2.9 (2.4–3.5)	0.005
BCAA, mmol/L	2.34 (1.92–2.74)	2.51 (1.91–2.91)	2.19 (1.63–2.67)	< 0.001
BCKA, *μ*mol/L	88.6 (74–105)	125.2 (117–132)	159.3 (146–172)	< 0.001
Current medication use, %				
Insulin	68 (57.0)	61 (51.0)	77 (65.0)	0.048
Metformin	54 (45.0)	46 (39.0)	44 (37.0)	0.377
AGI	45 (38.0)	50 (42.0)	37 (31.0)	0.060
DPP4i	20 (17.0)	31 (26.0)	33 (28.0)	0.403
SGLT2i	37 (31.0)	40 (34.0)	49 (41.0)	0.177
Statin	54 (45.0)	67 (56.0)	49 (41.0)	0.295
ACEI/ARB	42 (35.0)	39 (33.0)	33 (28.0)	0.729

**Table 3:** Correlation of BCAAs and BCKAs with thyroid hormone.

	**Model 1**		**Model 2**		**Model 3**	
	**b** ** (95% CI)**	**p** ** value**	**b** ** (95% CI)**	**p** ** value**	**b** ** (95% CI)**	**p** ** value**
T3						
BCAA	0.050 (0.010, 0.090)	0.015	0.045 (0.003, 0.086)	0.034	0.042 (0.002, 0.089)	0.045
BCKA	−0.002 (−0.003, −0.001)	< 0.001	−0.002 (−0.003, −0.001)	< 0.001	−0.002 (−0.003, −0.001)	< 0.001
T4						
BCAA	3.427 (0.748, 6.107)	0.012	3.435 (0.734, 6.136)	0.013	4.614 (1.384, 7.844)	0.005
BCKA	0.003 (−0.060, 0.066)	0.925	0.008 (−0.056, 0.071)	0.807	0.017 (−0.051, 0.085)	0.619
FT3						
BCAA	0.184 (0.072, 0.295)	0.001	0.149 (0.036, 0.262)	0.010	0.115 (0.016, 0.213)	0.023
BCKA	−0.004 (−0.007, −0.002)	0.002	−0.005 (−0.007, −0.002)	< 0.001	−0.004 (−0.006, −0.002)	< 0.001
FT4						
BCAA	0.589 (0.255, 0.923)	0.001	0.484 (0.148, 0.819)	0.005	0.430 (0.053, 0.807)	0.025
BCKA	−0.003 (−0.011, 0.005)	0.517	−0.005 (−0.013, 0.003)	0.248	−0.004 (−0.012, 0.004)	0.318

*Note:* In Model 1, independent variables included BCAAs or BCKAs. In Model 2, independent variables included age, gender, and BCAAs (or BCKAs). In Model 3, independent variables included variables in Model 2 plus duration of diabetes, HTN, SBP, DBP, smoking status, drinking status, FPG, HbA1c, HDL-C, BMI, and current medication use (including insulin, metformin, AGI, DPP4i, SGLT2i, statin, and ACER/ARB).

**Table 4:** Correlation of BCKA *Z*-scores with T3 and FT3 *Z*-scores.

	**Model 1**		**Model 2**		**Model 3**	
	**β** ** (95% CI)**	**p** ** value**	**β** ** (95% CI)**	**p** ** value**	**p** ** (95% CI)**	**p** ** value**
T3 *Z*-score						
BCKA *Z*-score	−0.194 (−0.301, −0.088)	< 0.001	−0.200 (−0.308, −0.091)	< 0.001	−0.192 (−0.299, −0.086)	< 0.001
FT3 *Z*-score						
BCKA *Z*-score	−0.162 (−0.265, −0.059)	0.002	−0.186 (−0.288, −0.083)	< 0.001	−0.156 (−0.260, −0.052)	< 0.001

*Note:* In Model 1, independent variables included BCKA *Z*-scores. In Model 2, independent variables included age, gender *Z*-scores, and BCKA *Z*-scores. In Model 3, independent variables included variables in Model 2 plus duration of diabetes *Z*-scores, HTN, SBP *Z*-scores, DBP *Z*-scores, smoking status, drinking status, FPG *Z*-scores, HbA1c *Z*-scores, HDL-C *Z*-scores, BMI *Z*-scores, and current medication use (including insulin, metformin, AGI, DPP4i, SGLT2i, statin, and ACER/ARB).

## Data Availability

The original data utilized in this study can be obtained from the corresponding authors upon reasonable request.

## Supporting Information

Supporting Information Additional supporting information can be found online in the Supporting Information section. Figure S1. Levels of thyroid function parameters according to tertiles of BCAAs and BCKAs. ⁣^∗^*p* < 0.05, ⁣^∗∗^*p* < 0.01, ⁣^∗∗∗^*p* < 0.001, T3 triiodothyronine, T4 thyroxine, FT3 free triiodothyronine, FT4 free thyroxine, BCAAs branched-chain amino acids, BCKAs branched-chain keto acids. Figure S2. Association of BCAAs and BCKAs with thyroid function parameters.
