# Supplementary material for: Association Between Thyroid Function Parameters and Plasma Branched-Chain Amino and Keto Acids in Patients With Euthyroid Type 2 Diabetes Mellitus
Source: J Diabetes Res. 2025 Jul 2;2025:2540444. doi: 10.1155/jdr/2540444 (PMC12240655; doi:10.1155/jdr/2540444)

**Supplementary materials**

**Supplementary** **Fig1**. levels of thyroid function parameters according to tertiles of BCAAs and BCKAs.

**p*<0.05, ***p*<0.01, ****p*<0.001,

T3 triiodothyronine, T4 thyroxine, FT3 free triiodothyronine, FT4 free thyroxine, BCAAs branched-chain amino acids, BCKAs branched-chain keto acids.


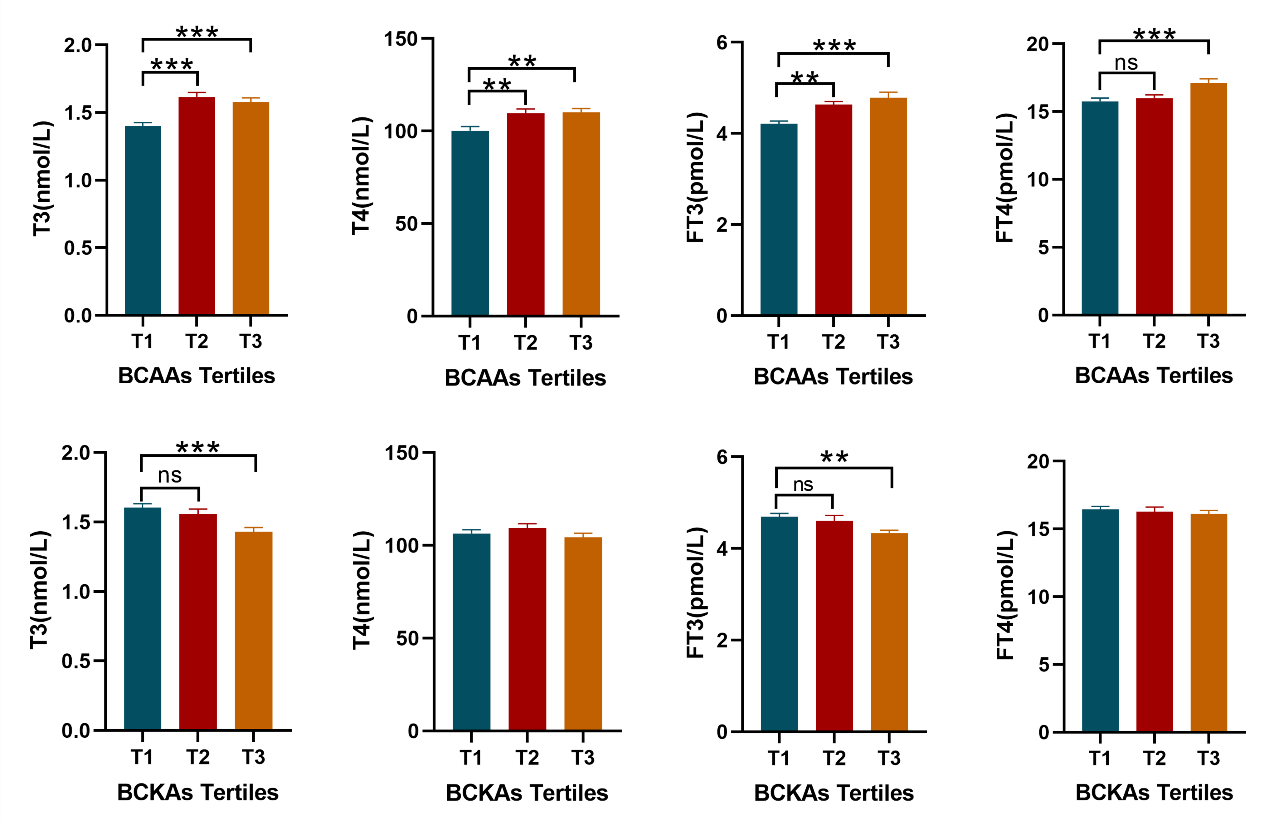


**Supplementary Fig2**. Association of BCAAs and BCKAs with thyroid function parameters.


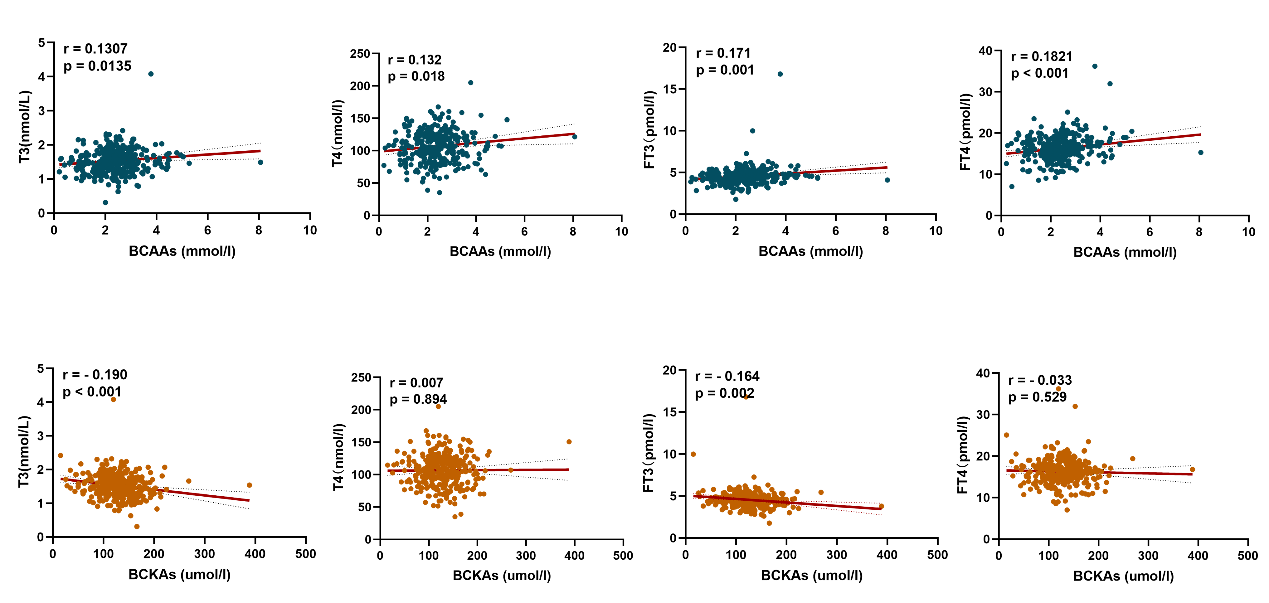

Supplement: Supporting Information — Additional supporting information can be found online in the Supporting Information section. Figure S1. Levels of thyroid function parameters according to tertiles of BCAAs and BCKAs. ⁣∗p < 0.05, ⁣∗∗p < 0.01, ⁣∗∗∗p < 0.001, T3 triiodothyronine, T4 thyroxine, FT3 free triiodothyronine, FT4 free thyroxine, BCAAs branched-chain amino acids, BCKAs branched-chain keto acids. Figure S2. Association of BCAAs and BCKAs with thyroid function parameters. [file 2540444.f1.docx]
